# 

**Published:** 1872-03

**Authors:** 


### BOOK NOTICES.

"Little Prudy's Flyaway Series." 1. Little Folks Astray. 2. Prudy Keeping House. 3. Aunt Madge's Story. These are published, and three more volumes to come, making a library of six volumes, illustrated. A good present for children, written by Sophie May, and published by Lee & Shepard, Boston, and Lee, Shepard & Dillingham, New York. The same house has issued "Ruby Duke," by Mrs. H. K. Potwin. 421 pp. 12mo. Also, "Among the Brigands," by Prof. Jas. De Mille. 328 pp. Illustrated.

"Edna Harrington; or, The Daughter's Influence in the Home Circle" (311 pp. 12mo), by the American Tract Society, 150 Nassau Street, New York, illustrated, is for old as well as young, at once interesting, amusing, and instructive. 90 cents. Also, "Tiptoe," by Katherine Williams. 283 pp. 80 cents. The writer makes an imperfect hero, and through his trials, temptations, and falls, endeavors to point one to the Saviour, as a personal friend, who is touched with the feeling of our infirmities. To be read by old and young with great profit. Also, "Pleasant Pictures," for young children, 126 pp., with a beautiful illustration on almost every other page, and in a style of binding well worth its price of eighty cents.

"Injurious Influences of School." Being a report by Dr. RUDOLPH VIRCHOW, Professor of Pathological Anatomy, at the University of Berlin; translated by John P. Jackson. A tract of 36 pages, for 25 cents, issued for the public good by the worthy enterprise of Miller, Haynes & Co., 21 West 26th Street, New York. This report should be carefully read by every parent who has children at school, and by every man and woman whose calling it is to impart instruction to the young. It shows how diseases of the eye are induced, congestions of the head, head-ache, bleeding at the nose, catarrh, curvature of the spine; diseases of the organs of the chest, lungs, heart, dyspepsias, contagious diseases, etc.

EYE DISEASES.

"Transactions of the American Ophthalmological Society," at their 8th Annual Meeting at Newport, Rhode Island, July, 1871. Among the "active members," of New York city, are Doctors Agnew, Delafield, Pomeroy, Loring, Curtis, and Norris. Interesting cases in relation to diseases of the eye were presented by B. Joy Jeffries, of Boston, Mass.; Dr. H. Knapp, 25 West 24th Street, New York city; Dr. Henry D. Noyes, do.; O. F. Wadsworth, Boston; Dr. Russell Murdock, Baltimore, Md.; Dr. George Strawbridge, Philadelphia, Pa.; Dr. John Green, St. Louis, Mo.; Dr. W. W. Seeley, Cincinnati, O. The names of the above are given purposely, that those of our readers having any ailment of the eye may know to what persons they may safely apply, and thus save us the trouble of answering letters from time to time. The eye and the ear are the most important and most delicate organs of the human body, and should be under the management of skillful, scientific men of high character; and we hereby take occasion to warn our readers against having anything whatever to do with peripatetic lecturers and wandering practitioners, in these diseases; if they were able men, they would have practice enough at home among the people who know them. The very fact of their having to advertise themselves in any way is proof positive that they are without skill and without marked ability, and too often they are adventurers and impostors. It is nearer the truth to say they are always so.

"Hand Book of Medical Microscopy," by JOSEPH D. RICHARDSON, M. D., Microscopist to the Pennsylvania Hospital. Published by J. B. Lippincott & Co., of Philadelphia and New York. This is a most important addition to the new science of Microscopy, which is opening up new worlds for observation. This volume commends itself to scientific men, and to the members of all the learned professions.

"London Lancet," monthly, $5 a year, postage 24 cents a year. The most authentic medical publication in the world. Address the agent, Wm. C. Herald, Esq., 52 John Street, New York.

"Harper's Weekly," with its 130,000 circulation, has an amazing popularity. "Harper's Bazar" gives an account of the fashions of the times for the ladies; and "Harper's Monthly," so highly prized by the educated classes, has a wider circulation than any other monthly magazine in the world; each are four dollars. They will be sent with "Hall's Journal of Health," during 1872, for $11 sent in a post-office order to Hurd & Houghton, 13 Astor Place, New York, thus saving $2.50 to the subscriber.

Morgan Envelope Company, Springfield, Mass., also manufacture paper in convenient form for writing letters, notes, legal cap manuscripts, printer's and reporter's copy; for authors, editors, students, reporters, and clergymen, with sermon covers and cases; not omitting the convenient glass gum pot, excluding dust and air. As this Journal is extensively read by all classes of educated people, many of whom, some time or other, write for papers and magazines, and as good articles have been written and never published, as have been published, and were not, because presented to the printer in an impracticable and faulty shape and arrangement, it is worth while to present their fourteen

DIRECTIONS FOR PROPERLY PREPARING MANUSCRIPT FOR THE PRESS.

1. Manuscript prepared for the press should never be written on both sides of the paper.

2. Write in so plain a hand that every word will be legible to the printer. Compositors prefer black ink.

3. Take particular care to make distinct every figure in numbers, and every letter in proper names.

4. Begin every paragraph one inch from the margin, or half an inch further from the margin than the lines that follow in the same paragraph.

5. Never begin a paragraph near the bottom of the page, but commence on another sheet.

6. Punctuate your manuscript as it ought to be printed, and leave half an inch space after every period.

7. If you want a word or sentence printed in *italics*, underscore it with one line; if in SMALL CAPITALS, with two lines; if in CAPITALS, with three lines.

8. Never depend upon the editor or printer to correct your manuscript.

9. If your article covers more than one sheet, be sure and number the pages in their order.

10. Repeat the title of your article or book, at the top of each page, on the same line with the number of the page. If the title is long, repeat only the first two or three words.

11. If you write more than one chapter, put a tape through the apertures and tie each chapter separately.

12. Never roll or fold your manuscript, but use envelopes as large as the sheet itself.

13. Any private communication to the editor should be written on a sheet by itself.

14. At the close of your article never fail to write the word "End," or signify the end by some mark.

By carefully observing the above rules, you will greatly facilitate type-setting, and make your manuscript much more acceptable to the editor: whereas good matter is sometimes rejected, in consequence of their non-observance.

"Essentials of the Principles and Practice of Medicine," by Prof. HARTSHORN, of Philadelphia, and published by Henry C. Lea, is a handbook of infinite practical value to both student and practitioner; its appreciation may be known by its having passed to its third edition, thoroughly revised. It brings us down to the close of the last year in its handling of the latest topics, as tuberculosis, relapsing fever, carbolic acid, hydrate of chloral, the error and danger of indiscriminate stimulism, and other important subjects.

"Handbook of Therapeutics," by SIDNEY RINGER, M. D., Professor of Therapeutics in University College and Hospital, Philadelphia, is just published by Wm. Wood & Co., 27 Great Jones Street, New York. 483 pages. This is a succinct and practical volume. A striking feature is to state the symptom or group of symptoms which may suggest a medicine and the way of its administration without taking up time in explaining how the medicine effects a cure. It contains a very valuable and convenient Alphabetical Table, showing the amount or dose of each medicine, and a not less valuable dietary for invalids, how to prepare the different kinds of food for the sick, with a copious index of the medicines treated of. The book is just issued, and we shall be surprised if the intelligence of the profession shall not call for a second or even third edition before the close of the year in which the first is issued, because it is a severe want of the time.

"Hearth and Home." Take it to your home. It is so much the fashion nowadays to convey information, and moral truths and sentiments, in the form of stories, that even some popular lecturers have adopted this style of address. The mass of people, especially the young, demand stories to such a degree, that papers filled with sensational novels and exciting, trashy stuff, have a wide circulation. To forestall this taste, and supply something better to the masses, the Publishers of "Hearth and Home," in addition to the usual variety of that paper, have engaged a corps of first-class writers, among whom are Jean Ingelow, Edward Eggleston, Mary E. Dodge, Louisa M. Alcott, Edward Everett Hale, Elizabeth Stuart Phelps, Harriet Prescott Spofford, Rose Terry, Maria R. Oakey, Lucia G. Runkle, and many others, who furnish to this Journal the *best Original Stories,* of the *purest character* and *highest grade* — thus conveying much instruction in a pleasing form. Besides these, the weekly "Hearth and Home" contains a large amount of first-class reading, editorials, literature, art, science, amusement; instruction for the housekeeper, the gardener, the farmer; a capital department for Children and Youth; the news of the day; financial and market reports, etc. Its engravings cost over $25,000 a year. "Hearth and Home" is such a journal as may be safely and very profitably taken into any family. It is supplied at the low rate of $3 a year; Orange Judd & Co., Publishers, 245 Broadway, New York city.

"Anderson's Historical Reader," published by Clark & Maynard, No. 5 Barclay Street, New York (544 pages), like all the school histories of Anderson, is well worthy of the attention of school committees and public and private teachers. The selections are made with great care, not only as to their appropriateness, but as to their authenticity, to which point it is to be hoped a constantly growing attention should be paid in every school book published.

